# Association of low-calorie sweetened product consumption and intakes of free sugar and dietary patterns in UK adults: a national study from 2008 to 2019

**DOI:** 10.3389/fnut.2026.1797836

**Published:** 2026-05-14

**Authors:** Mathuramat Seesen, Kiara Chang, Christopher Millett, Fernanda Rauber, Renata B. Levy, Mathilde Touvier, Edward W. Gregg, Eszter P. Vamos

**Affiliations:** 1 Public Health Policy Evaluation Unit, School of Public Health, Imperial College London, London, United Kingdom; 2 Public Health Research Centre and Comprehensive Health Research Centre, NOVA National School of Public Health, NOVA University Lisbon, Lisbon, Portugal; 3 Centre for Epidemiological Studies in Health and Nutrition (NUPENS), University of São Paulo, São Paulo, Brazil; 4 Department of Preventive Medicine, School of Medicine, University of São Paulo, São Paulo, Brazil; 5 Institute of Biomedical Research of Salamanca (IBSAL), University of Salamanca, Salamanca, Spain; 6 Université Sorbonne Paris Nord and Université Paris Cité, INSERM, INRAE, CNAM, Center of Research in Epidemiology and StatisticS (CRESS), Nutritional Epidemiology Research Team (EREN), Bobigny, France; 7 Department of Epidemiology and Biostatistics, School of Public Health, Imperial College London, London, United Kingdom; 8 School of Population Health, RCSI University of Medicine and Health Science, Dublin, Ireland

**Keywords:** dietary patterns, dietary trends, free sugar intake, low- and no-calorie sweetened products, UK adults, ultra-processed foods

## Abstract

**Introduction:**

Evidence on the effects of low- and no-calorie sweetened (LCS) product consumption on overall dietary patterns remains limited, despite their frequent promotion as healthier alternatives. This study assessed the association between LCS product consumption and both the levels and temporal trends in free sugar intake and overall dietary patterns among UK adults.

**Methods:**

Annual cross-sectional data from the National Diet and Nutrition Survey (NDNS) from 2008/09 to 2018/19 were analyzed. Adults aged ≥18 years (*N* = 8,304) were categorized into four levels of LCS product consumption: non-consumers (No-LCS) and three tertiles of consumers (Low-LCS: ≤75.0 g/day, Mid-LCS: >75.0–216.8 g/day, and High-LCS: >216.8 g/day), as measured by four-day food diaries. The associations between LCS product consumption and intakes of free sugar, energy, and minimally processed and ultra-processed foods as determined by the Nova classification were analyzed using multivariable linear regression, adjusted for sociodemographic factors.

**Results:**

In 2008/09, 45.9% of participants consumed LCS products, and this proportion remained stable over the 11-year period. Among LCS product consumers, the median LCS product intake increased significantly from 132.0 g/d in 2008/09 to 170.0 g/d in 2018/19. In the No-LCS group, free sugar and energy intakes decreased by −1.0 g/d (95% CI, −1.4 to −0.6) and −6.7 kcal/d (95% CI, −11.4 to −2.1) per year, respectively, with similar declines observed for the High-LCS group. In 2008/09, the mean water and minimally processed food consumption was lower in the High-LCS than the No-LCS group, by −141.2 g/d (95% CI, −203.8 to −78.6) and −306.3 g/d (95% CI, −359.5 to −216.7), respectively, and this difference persisted throughout the study period.

**Discussion:**

LCS consumption does not appear to align with potentially beneficial dietary patterns regarding free sugar, ultra-processed food and beverage, and minimally processed food and beverage intakes. These findings underline the importance of policies to consider overall dietary patterns that also address LCS consumption alongside reducing free sugar intake.

## 1 Introduction

Obesity and diet-related ill health are critical public health challenges globally and in the UK. Within Europe, the UK has one of the highest obesity rates, with approximately two in three adults being classified as obese or overweight (1). As excessive consumption of free sugar, defined as sugar added to food as well as those naturally present in honey, syrup, and unsweetened fruit juices (2), has been identified as a significant contributing factor to the rising burden of obesity, a broad range of policies have placed a significant focus on reducing their intake (3). These policies have encouraged reductions in sugar intake and substitution away from sugar-sweetened products toward alternatives including “sugar-free” or “no added sugar” products (4, 5), which in the current food environment often contain low- and no-calorie sweeteners (LCS) (6).

In this study, we defined LCS products as foods and beverages that contain low- or no-calorie sweeteners, or sugar alcohols. Some of these sweeteners are commonly referred to in the literature as non-nutritive sweeteners, non-sugar sweeteners, or non-caloric sweeteners (7, 8). In the UK, commonly used LCS include acesulfame K, aspartame, saccharin, steviol glycosides, and sucralose, as well as sugar alcohols such as erythritol, sorbitol, and xylitol (9). Although data on the consumption of LCS products are limited, pooled data from 2008 to 2012 indicate that UK adults consumed an average of 108 g/day LCS soft drinks, compared with 136 g/day for sugar-sweetened soft drinks (10). Between 2015 and 2018, sales of LCS soft drinks increased by 40% in the UK, alongside a parallel decline in the sales of sugar-sweetened beverages (SSBs) (11). Similar trends have been observed in other high-income countries, where per capita sales of added sugar in beverages decreased while the non-nutritive sweeteners from beverage sales increased between 2007 and 2019 (12).

Despite an intense policy focus on sugar reduction, recent data on the relationship between LCS product consumption and free sugar and energy intake in the UK populations remain limited (13, 14). Moreover, while free sugar intake among UK adults has decreased over the past decade, consumption remains well above the recommended limit of 5% of total energy intake, as set by the UK Scientific Advisory Committee on Nutrition (SACN) and the World Health Organization (15, 16).

Relatedly, in recent years, there has been a surge in evidence connecting ultra-processed foods (UPFs) to obesity and poor health, raising global concerns (17). The UK is among the highest consumers of UPFs in Europe, with UPFs accounting for more than half of the daily energy intake in the UK population (18). The emergence of the UPF concept has shifted focus toward broader characteristics of foods and dietary patterns that complements the well-established nutrient-centric recommendations for healthy diet (19). Alongside the intensifying policy discourse around UPFs (20), concerns have been raised about the increasing popularity of LCS products, which are also classified as UPFs, and their promotion as healthy options (21). However, evidence on the association between LCS consumption and free sugar intake remains limited, with mixed findings (13, 14, 22, 23), and its relationship with broader dietary patterns based on the degree and purpose of food processing has been examined in only one study conducted in UK children (24), with no comparable evidence available in adult populations.

In 2023, the World Health Organization published a guideline recommending against the use of non-sugar sweeteners for weight control and for reducing the risk of diet-related non-communicable diseases (7). In contrast, the UK’s Scientific Advisory Committee on Nutrition recently suggested that non-sugar sweeteners may help reduce sugar intake and support short-term weight management, although they are not essential and should be limited (21).

Given the high consumption of ultra-processed foods and the pressing challenge of obesity in the UK population, it is important to understand the association between LCS product consumption and overall dietary patterns. To address this knowledge gap, this study aimed to examine the temporal trends and associations between LCS product consumption and intakes of free sugar as well as broader dietary patterns characterized by minimally processed food and UPF consumption, using nationally representative data of UK adults from 2008 to 2019.

## 2 Materials and methods

### 2.1 Data source and study population

This study utilized data from adults (18 years of age and above) participating in the National Diet and Nutrition Survey (NDNS), a serial annual-rolling cross-sectional survey nationally representative of the UK population, from year 1 (2008–2009) to year 11 (2018–2019) (25). Information regarding the methodology of the NDNS is available elsewhere (26). In summary, individuals living in private households were enrolled using stratified random sampling.

Participants’ socio-demographic data were collected through computer assisted personal interviews. Dietary consumption was assessed using a four-day food diary, and the four consecutive days were computer-selected to ensure the survey provides good representativeness from all days of the week (27). Participants were asked to record all foods and beverages they consumed including the portion size, brand names, and any additional ingredient or condiments used (e.g., fats, sugar, sweeteners, and sauces). For homemade foods, cooking methods, ingredients used (including their quantities) and portion sizes were also recorded. Nutritional intakes were obtained by linking dietary consumption data to the year-specific NDNS Nutrient Databank, which is updated annually to reflect changes in the UK food supply and the nutrient composition of foods (28).

We excluded a total of 24 adult participants with an average energy intake outside of 500–5,000 kcal/day, as these values were considered implausible for habitual daily dietary intake (29). We further excluded 11 adults with missing ethnicity data and 8 adults with missing Index of Multiple Deprivation (IMD) data. Overall, 8,304 adults pooled across the 11-year period were included in the final analytical sample.

### 2.2 Study exposure

We systematically identified LCS products based on sweeteners in the UK approved additives list (30). Among the 5,196 food and beverage items reported as consumed by NDNS participants, ingredient lists were searched for branded products, while descriptors and ingredient information were used to classify generic items. In total, 140 items were classified as LCS products, including 53 foods and 87 LCS beverages. The details in the process of identifying LCS products in the NDNS data are presented in Supplementary Figure 1.

All participants recorded their food intake for either 3 days (*n* = 177) or 4 days (*n* = 8,127). We calculated the daily LCS product consumption for each adult by taking an average of the total quantity (in grams) of LCS products consumed over multiple days and expressed in g/day. Participants were further categorized into four groups based on their daily LCS product consumption: the No-LCS, Low-LCS, Mid-LCS, and High-LCS groups. Participants who consumed 0 g/d of LCS products were categorized as the No-LCS group. The cutoff values for LCS groups among consumers were derived based on the tertile of LCS product consumption among survey respondents participated in the year 2008–2009. This is a common approach used by nutritional epidemiological studies and ensures all LCS groups have similar sample sizes for comparison (31, 32). Those who consumed LCS products at ≤ 75.0 g/d, > 75.0–216.8 g/d, and > 216.8 g/d, were classified as Low-LCS, Mid-LCS, and High-LCS groups, respectively.

### 2.3 Outcomes

The primary study outcome is daily intake of free sugar, defined as all sugars added to foods by manufacturers or in food preparation, as well as sugars naturally present in honey, syrups, nectars, and unsweetened fruit juices (2). Secondary outcomes include daily intakes of total energy, total sugar, water, minimally processed food and beverages, ultra-processed foods, and ultra-processed beverages. The minimally processed and ultra-processed foods and beverages were identified using the Nova food classification system which assigns foods into one of the four groups based on the extent and purpose of food processing they undergo (33). The classification was conducted manually by researchers with extensive experience in applying NOVA to large-scale dietary datasets, with internal discussion of ambiguous cases to ensure consistency in decision-making. Classification was conducted manually and independently by two trained nutritional epidemiologists. The detailed Nova classification used for NDNS foods has been published previously (18).

Nova 1 includes unprocessed and minimally processed foods and beverages that have undergone simple processing methods or treatments to make them edible, such as squeezing, freezing, or pasteurization, without the addition of salt, sugar, or oils. Examples are fresh fruits, vegetables, red meat and poultry, grains, and plain milk. Nova 2 refers to processed culinary ingredients used for seasoning and cooking. They are derived from Nova 1 foods or natural sources, and include sugar, salt, vegetable oils, and butter. Nova 3 encompasses processed foods and beverages created by adding salt, sugar, oil, or other Nova 2 ingredients to Nova 1 foods. Examples include cured meats, canned fish, artisanal cheeses and bread. Nova 4 represents ultra-processed foods and beverages, which are formulations of ingredients derived from extensive industrial processing. Examples include packaged snacks, flavored milk or yogurt, confectioneries, mass-produced bakery products, margarine, breakfast cereals containing food additives, carbonated drinks, and LCS products.

Total energy intake was derived by the average of total kilocalories consumed across multiple days (kcal/d). All other dietary variables were expressed as the average intake in grams over multiple recorded days (g/d). We also assessed energy from free sugar as a percentage of total energy intake (%kcal/d) to provide contextual comparison with UK and WHO recommended limits of less than 5% of total energy intake (15, 16). The consumption of ultra-processed and minimally processed foods and beverages were additionally assessed in kilocalories averaged over multiple recorded days (kcal/d).

### 2.4 Covariates

The socio-demographic variables in this study included age, sex (men, women), ethnicity (white, non-white), equivalized household income tertiles (high, middle, low), IMD (quintile 1 (most deprived) to 5 (least deprived)), body mass index (BMI, normal and underweight, overweight, obese), and smoking status (current smoker, ex-smoker, never smoked). IMD is a measure of relative deprivation for small areas across the UK. It is based on seven domains, namely, income, employment, education, health, crime, barriers to housing and services, and living environment (34). BMI data, derived from objectively measured weight and height, were available. Participants with BMI < 25 kg/m^2^, 25 to < 30 kg/m^2^, and ≥ 30 kg/m^2^ were categorized into three groups: normal and underweight, overweight, and obese, respectively.

### 2.5 Statistical analysis

Participants’ characteristics across LCS consumption levels within each year were compared using Kruskal-Wallis test for continuous variables and Chi-square test for categorical variables as appropriate. Additionally, a comparison between the characteristics of participants between the years 2008–2009 and 2018–2019 was conducted using Wilcoxon rank sum test for continuous and chi-square test for categorical variables.

The overall dietary pattern for each LCS group is presented in bar graphs showing the mean percentages of daily food/energy intake contributed by each Nova subgroup. The association between LCS product consumption and each study outcome was examined using multivariable linear regression. The model included, apart from the LCS consumption group and year of survey (continuous) variables, an interaction term between those to assess the variation in trends of study outcome across LCS consumption groups. Multiple Imputation by Chained Equations, using 20 imputed copies, was employed to impute missing data for household income (14.6%), BMI (7.8%), and smoking status (0.04%). Additionally, all covariates and NDNS survey weight were adjusted for in the regression models. Bonferroni correction was applied to reduce the risk of type I error arising from multiple comparisons. All analyses were carried out using R software version 4.2.2. The *p*-value reported were two-tailed, and statistical significance was defined as a p-value below 0.05.

## 3 Results

A total of 8,304 adults were included in the analysis over the 11-year study period from 2008–2009 to 2018–2019 following an exclusion of 24 adults identified with an implausible daily energy intake, with the number of participants ranging from 585 to 1,084 across years. Table 1 presents the characteristics of participants by levels of LCS product consumption in the first and final study years. In 2008–2009, 45.9% of participants consumed LCS products, and this proportion varied between 47.2 and 57.8% over the 11-year period (Supplementary Figure 2). The median of LCS product consumption among consumers increased significantly from 132.0 g/d in 2008–2009 to 170.0 g/d in 2018–2019. This overall increase was solely attributable to the rise in consumption in the High-LCS group (Supplementary Table 1).

**Table 1:** Characteristics of adults in the National Diet and Nutrition Survey in 2008–2009 (*N* = 836) and 2018–2019 (*N* = 585) according to levels of consumption of low- and no-calorie sweetened products.

Characteristics	NDNS year 2008–2009 (*n* = 836)						NDNS year 2018–2019 (*n* = 585)						2008–2009 vs. 2018–2019
	Total (*n* = 836)	No-LCS (*n* = 452)	Low-LCS (*n* = 141)	Mid-LCS (*n* = 115)	High-LCS (*n* = 128)	*P*-value	Total (*n* = 585)	No-LCS (*n* = 297)	Low-LCS (*n* = 81)	Mid-LCS (*n* = 82)	High-LCS (*n* = 125)	*P*-value	*P*-value
Age, median (IQR)	46.5 (28.0)	51.0 (27.0)	45.0 (25.0)	45.0 (24.5)	38.0 (21.3)	<0.001^+^	50.0 (29.0)	53.0 (30.0)	55.0 (31.0)	45.5 (26.0)	44.0 (25.0)	<0.001^+^	0.06
Men, *n* (%)	355 (42.5)	216 (47.8)	44 (31.2)	47 (40.9)	48 (37.5)	<0.01**	244 (41.7)	121 (40.7)	37 (45.7)	36 (43.9)	50 (40.0)	0.8	0.8
Ethnic group, *n* (%)						0.1						<0.01**	0.1
White	786 (94.0)	424 (93.8)	132 (93.6)	104 (90.4)	126 (98.4)		535 (91.5)	259 (87.2)	76 (93.8)	78 (95.1)	122 (97.6)		
Non-white	50 (6.0)	28 (6.2)	9 (6.4)	11 (9.6)	2 (1.6)		50 (8.5)	38 (12.8)	5 (6.2)	4 (4.9)	3 (2.4)		
Household income tertile, *n* (%)						0.2						0.3	0.9
Highest	259 (31.0)	132 (29.2)	46 (32.6)	37 (32.2)	44 (34.4)		175 (29.9)	77 (25.9)	28 (34.6)	31 (37.8)	39 (31.2)		
Middle	246 (29.4)	133 (29.4)	40 (28.4)	42 (36.5)	31 (24.2)		168 (28.7)	83 (27.9)	23 (28.4)	24 (29.3)	38 (30.4)		
Lowest	219 (26.2)	117 (25.9)	35 (24.8)	29 (25.2)	38 (29.7)		158 (27.0)	85 (28.6)	19 (23.5)	18 (22.0)	36 (28.8)		
Missing value	112 (13.4)	70 (15.5)	20 (14.2)	7 (6.1)	15 (11.7)		84 (14.4)	52 (17.5)	11 (13.6)	9 (11.0)	12 (9.6)		
IMD, *n* (%)						0.04*						0.9	0.07
Quintile 1	171 (20.5)	95 (21.0)	29 (20.6)	23 (20.0)	24 (18.8)		103 (17.6)	58 (19.5)	15 (18.5)	12 (14.6)	18 (14.4)		
Quintile 2	161 (19.3)	99 (21.9)	23 (16.3)	20 (17.4)	19 (14.8)		122 (20.9)	62 (20.9)	14 (17.3)	20 (24.4)	26 (20.8)		
Quintile 3	152 (18.2)	73 (16.2)	24 (17.0)	22 (19.1)	33 (25.8)		128 (21.9)	62 (20.9)	14 (17.3)	20 (24.4)	32 (25.6)		
Quintile 4	184 (22.0)	98 (21.7)	34 (24.1)	17 (14.8)	35 (27.3)		109 (18.6)	53 (17.8)	20 (24.7)	13 (15.9)	23 (18.4)		
Quintile 5 (least deprive)	168 (20.1)	87 (19.2)	31 (22.0)	33 (28.7)	17 (13.3)		123 (21.0)	62 (20.9)	18 (22.2)	17 (20.7)	26 (20.8)		
BMI, mean (SD)	27.4 (5.4)	27.0 (5.2)	27.7 (4.9)	27.7 (5.6)	28.7 (6.4)	0.02*	27.1 (5.4)	26.3 (5.3)	27.2 (5.8)	25.6 (3.7)	30.0 (5.4)	<0.001^+^	0.3
Smoking status, *n* (%)						0.03*						0.3	<0.001^+^
Current smoker	195 (23.3)	112 (24.8)	26 (18.4)	19 (16.5)	38 (29.7)		104 (17.8)	51 (17.2)	9 (11.1)	14 (17.0)	30 (24.0)		
Ex-smoker	196 (23.4)	101 (22.4)	39 (27.7)	36 (31.3)	20 (15.6)		127 (21.7)	69 (23.2)	14 (17.3)	17 (20.7)	27 (21.6)		
Never smoked	445 (53.2)	239 (52.9)	76 (53.9)	60 (52.2)	70 (54.7)		328 (56.1)	161 (54.2)	53 (65.4)	49 (59.8)	65 (52.0)		
Missing	0 (0)	0 (0)	0 (0)	0 (0)	0 (0)		26 (4.4)	16 (5.4)	5 (6.2)	2 (2.4)	3 (2.4)		

*P-value < 0.05, **P-value < 0.01, ^+^*P*-value < 0.001. Statistical analysis: Kruskal-Wallis test was used to compare the distribution of age and BMI across different consumption levels of LCS products. Wilcoxon rank sum test was used to compare age and BMI between year 1 and year 11. Chi-square test was used to compare the proportions of categorical variables across different consumption levels of LCS products and between year 1 and year 11. NDNS, National Diet and Nutrition Survey; LCS products, no- and low-calorie sweetened products; IQR, interquartile range; IMD, Index of Multiple Deprivation; BMI, body mass index. Levels of LCS product consumption: No-LCS, no LCS product consumption (0 gram/day); Low-LCS, daily LCS product consumption ≤ 75.0 g; Mid-LCS, daily LCS product consumption > 75.0–216.8 g; High-LCS, average daily LCS product consumption >216.8 g.

In 2008–2009, the median of participants’ age was 46.5 years (IQR: 28.0) and mean BMI was 27.4 (SD 5.4) kg/m^2^ (Table 1). The greatest proportion of participants were women (57.5%), with white ethnicity (94.0%), were in the highest household income tertile (31.0%), and had never smoked (53.2%). The characteristics of participants remained similar from 2008–2009 to 2018–2019 except for smoking status where a smaller proportion of participants were current smokers in 2018–2019.

Compared across LCS consumption groups, participants in the higher LCS consumption groups were younger and had a higher mean BMI in both 2008–2009 and 2018–2019. The proportion of women and those never smoked were larger among groups with higher LCS consumption in 2008–2009. However, these differences were no longer present in 2018–2019.

**Figure 1:**
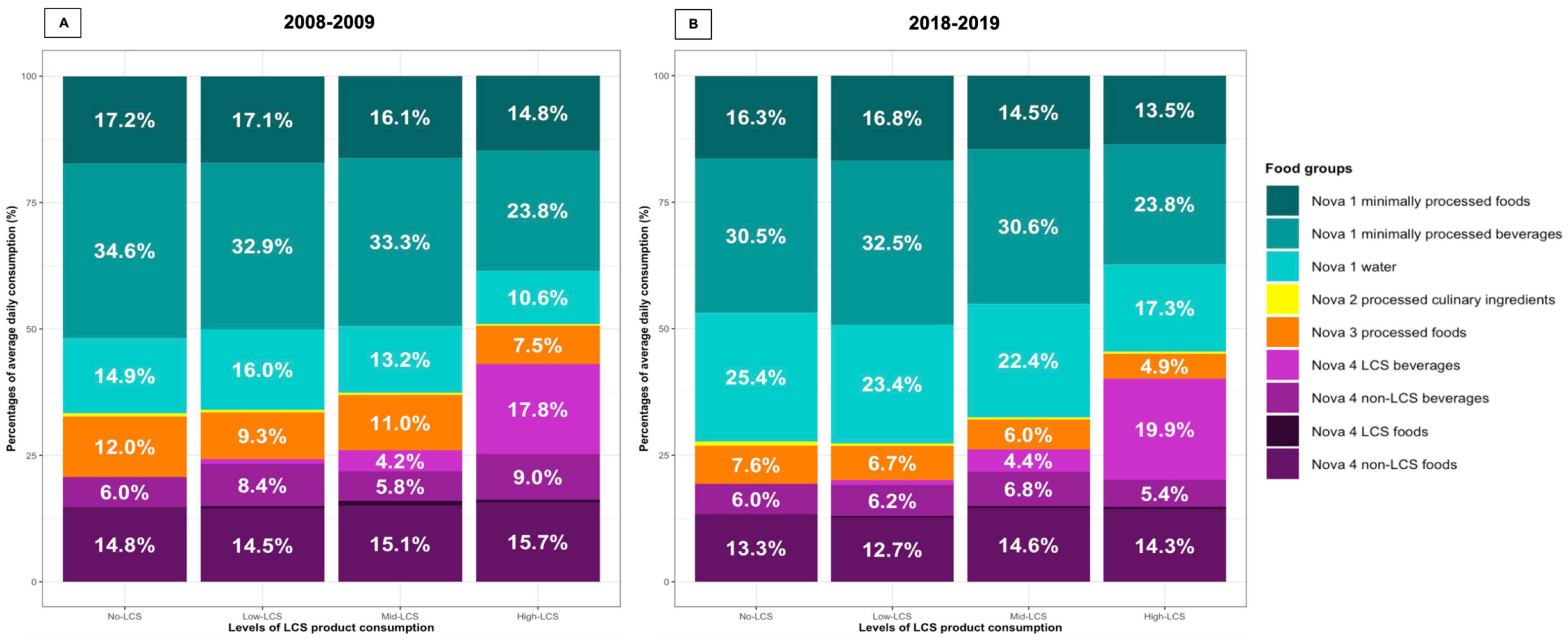
Dietary patterns by NOVA subgroups among UK adults according to the levels of no and low-calorie sweetened products in 2008–2009 **(A)** and 2018–2019 **(B)**. **(A)** (*N* = 836). Percentages of dietary intake by NOVA subgroups. No-LCS: NOVA 2 processed culinary ingredients, 0.6%. Low-LCS: NOVA 2 processed culinary ingredients, 0.5%; NOVA 4 LCS beverages, 0.9; NOVA 4 LCS foods, 0.5%. Mid-LCS: NOVA 2 processed culinary ingredients, 0.5%; NOVA 4 LCS foods, 0.9%. High-LCS: NOVA 2 processed culinary ingredients, 0.4%; NOVA 4 LCS foods, 0.5%. **(B)** (*N* = 585). Percentages of dietary intake by NOVA subgroups. No-LCS: NOVA 2 processed culinary ingredients, 0.8%. Low-LCS: NOVA 2 processed culinary ingredients, 0.5%; NOVA 4 LCS beverages, 1.0; NOVA 4 LCS food, 0.2%. Mid-LCS: NOVA 2 processed culinary ingredients, 0.4%; 1.7%; NOVA 4 LCS foods, 0.3%. High-LCS: NOVA 2 processed culinary ingredients, 0.4%; NOVA 4 LCS foods, 0.4%. Abbreviations: LCS product, no- and low-calorie sweetened product; LCS foods, low-calorie sweetened foods; LCS beverages, low-calorie sweetened beverages. Levels of LCS product consumption: No-LCS, no LCS product consumption (0 gram/day); Low-LCS, daily LCS product consumption ≤ 75.0 g; Mid-LCS, daily LCS product consumption >75.0–216.8 g; High-LCS, average daily LCS product consumption >216.8 g.

Figure 1 shows the mean intake of each Nova subgroup expressed as a percentage of total daily food and beverage intake by LCS consumption groups in 2008–2009 (Figure 1A) and 2018–2019 (Figure 1B). In 2008–2009, the High-LCS group had a lower consumption of minimally processed foods and beverages including water, but a higher consumption of non-LCS ultra-processed foods and beverages compared with the other groups. This pattern remained similar in 2018–2019 for the consumption of minimally processed foods and beverages despite an increase in water consumption in all LCS groups. The consumption of ultra-processed foods and beverages in 2018–2019 remained at similar levels to 2008–2009 in all LCS groups. However, the consumption of non-LCS ultra-processed foods and beverages reduced in the High-LCS group that complements an increase in LCS ultra-processed beverages in 2018–2019. When dietary patterns were assessed instead by the proportion of daily energy intake, there was a gradient of increasing dietary energy obtained from ultra-processed foods and beverages with higher levels of LCS product consumption in both 2008–2009 and 2018–2019 (Supplementary Figure 3).

### 3.1 Free sugar, total sugar and energy intake from 2008–2009 to 2018–2019

**Figure 2:**
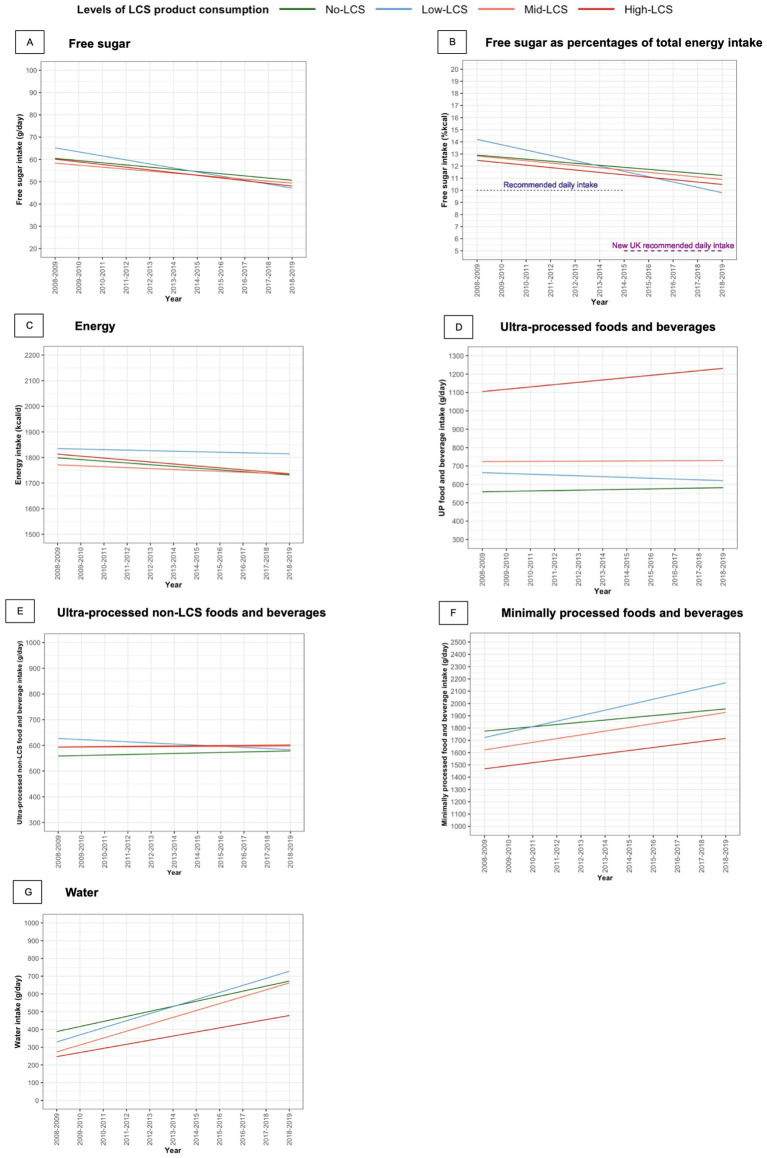
Trends in intakes of free sugar, energy, and dietary components by levels of LCS product consumption among UK adults. LCS product, no- and low-calorie sweetened product; kcal, kilocalories. Levels of LCS product consumption: No-LCS, no LCS product consumption (0 gram/day); Low-LCS, daily LCS product consumption ≤ 75.0 g; Mid-LCS, daily LCS product consumption >75.0–216.8 g; High-LCS, average daily LCS product consumption >216.8 g.

Multivariable linear regression analysis showed no significant difference in free sugar intake between the No-LCS group and other groups in 2008–2009 after Bonferroni correction (Table 2; Figure 2). Over the 11-year period, free sugar intake in the No-LCS group declined by −1.0 g/d (95% CI, −1.4 to −0.6) annually. Similar declines were observed for the Mid-LCS and High-LCS groups compared with the No-LCS group, while a greater decline was evident in the Low-LCS group. By 2018–2019, all LCS consumer groups had similar levels of free sugar intake as the No-LCS group (Supplementary Table 3).

**Table 2:** Association between daily LCS product consumption and intakes of energy, sugar, and dietary components from 2008 to 2019 (*n* = 8,304).

Intake of nutrients	Free sugar intake (g/d)	Free sugar intake (%kcal/d)	Total energy intake (kcal/d)	Ultra-processed food and beverage intake (g/d)	Ultra-processed non-LCS food and beverage intake (g/d)	Minimally processed food and beverage intake (g/d)	Water intake (g/d)
	Coefficient (95% CI)	Coefficient (95% CI)	Coefficient (95% CI)	Coefficient (95% CI)	Coefficient (95% CI)	Coefficient (95% CI)	Coefficient (95% CI)
LCS product consumption at year 1^+^							
No-LCS	References	References	References	References	References	References	References
Low-LCS	4.7 (0.1, 9.3)*	1.3 (0.6, 2.0)*^+^	36.2 (−22.2, 94.5)	104.3 (59.8, 148.9)*^+^	68.4 (28.7, 108.0)*	−52.0 (−141.8, 37.8)	−58.5 (−121.2, 4.2)
Mid-LCS	−2.1 (−7.1, 2.8)	−0.1 (−0.9, 0.7)	−27.1 (−90.4, 36.1)	164.5 (116.1, 212.8)*^+^	34.3 (−8.6, 77.3)	−152.2 (−249.5, −54.9)*	−114.5 (−182.4, −46.5)*
High-LCS	−0.3 (−4.9, 4.2)	−0.4 (1.2, −0.04)	14.0 (−44.3, 72.3)	545.3 (500.8, 589.8)*^+^	35.0 (−4.5, 74.6)	−306.3 (−395.9, −216.7)*^+^	−141.2 (−203.8, −78.6)*^+^
Survey year	−1.0 (−1.4, −0.6)*^+^	−0.2 (−0.2, −0.1)*^+^	−6.7 (−11.4, −2.1)*	2.2 (−1.3, 5.8)	2.0 (−1.2, 5.2)	18.2 (11.0, 25.3)*^+^	28.4 (23.4, 33.5)*^+^
Interaction							
No-LCS × Year	References	References	References	References	References	References	References
Low-LCS × Year	−0.8 (−1.6, −0.03)*^+^	−0.3 (−0.4, −0.1)*^+^	4.7 (−5.3, 14.8)	−6.6 (−14.3, 1.1)	−6.3 (−13.2, 0.5)	26.4 (11.0, 41.9)*	11.4 (0.6, 22.2)*
Mid-LCS × Year	0.1 (−0.8, 0.9)	−0.03 (−0.2, 0.1)	3.0 (−7.7, 13.7)	−1.6 (−9.8, 6.5)	−1.6 (−8.8, 5.7)	12.3 (−4.0, 28.7)	10.4 (−1.1, 21.9)
High-LCS × Year	−0.2 (−1.0, 0.5)	−0.03 (−0.1, 0.1)	−0.9 (−10.3, 8.5)	10.4 (3.3, 17.6)*	−1.2 (−7.6, 5.2)	6.6 (−7.8, 21.0)	−5.3 (−15.4, 4.8)

*p-value < 0.05 before Bonferroni correction, *^+^*p*-value < 0.05 after Bonferroni correction. Statistical analysis: Multivariable linear regression adjusted for age, sex, ethnicity, household income, Indices of Multiple Deprivation, body mass index, and smoking status. LCS product consumption at Year 1: Since the Survey year variable was centered at Year 1, these estimates provide the average difference in outcome for each LCS consumption group compared with the No-LCS group in 2008–2009 (Year 1); Survey year: represents the trend or annual change in outcome over time for the No-LCS group; Interaction term (LCS consumption group × survey year): assesses the difference in trends for each LCS consumption group compared with the No-LCS group. kcal, kilocalories LCS product, no- and low-calorie sweetened product. Levels of LCS product consumption: No-LCS, no LCS product consumption (0 gram/day); Low-LCS, daily LCS product consumption ≤ 75.0 g; Mid-LCS, daily LCS product consumption >75.0–216.8 g; High-LCS, average daily LCS product consumption >216.8 g.

When expressed as a percentage of daily energy intake (%kcal/d), free sugar intake was higher in the LCS groups compared with the No-LCS group by 1.3%kcal/d (95% CI, 0.6 to 2.0) in 2008–2009 (Table 2; Figure 2). Trends over time were similar to those observed for free sugar intake expressed in grams. In 2018–2019, the Low-LCS group had a lower percentage of daily energy intake from free sugar in 2018–2019 (Supplementary Table 3).

Despite the decline in free sugar intake, these levels remained two-fold above the UK and WHO recommended limits throughout the 11-year study period (Figure 2). Results for total sugar intake were consistent with those observed for free sugar intake (Supplementary Tables 2, 3).

The total energy intake did not differ significantly between LCS groups in 2008–2009. Although similar declines in energy intake were observed over the 11-year study period in all LCS groups, this did not remain significant after Bonferroni correction (Table 2).

### 3.2 Ultra-processed food and beverage intake from 2008–2009 to 2018–2019

In 2008–2009, all groups of LCS consumers had a higher consumption of ultra-processed food and beverages compared with the No-LCS group. However, there was no difference in the consumption of ultra-processed non-LCS foods and beverages between the No-LCS group and the other groups (Table 2). Over the 11-year study period, there was no evidence of a significant change in the consumption of ultra-processed foods and beverages in the No-LCS group, Low-LCS, or Mid-LCS groups. Although the High-LCS group showed a significant and upward trend compared with the No-LCS group, this association no longer reached statistically significant after Bonferroni correction (Table 2; Figure 2).

The daily energy intake from ultra-processed foods and beverages were found higher in all LCS consumer groups compared with the No-LCS group in 2008–2009, but these were not statistically significant after Bonferroni correction except for the High-LCS group (Supplementary Tables 2, 4). Moreover, the daily energy intake from ultra-processed foods and beverages did not significantly change over the 11-year study period, and this was similar across all LCS groups (Supplementary Table 2).

### 3.3 Intakes of water and minimally processed foods and beverages from 2008–2009 to 2018–2019

In 2008–2009, the High-LCS groups had a significantly lower intake of water and minimally processed foods and beverages, by −141.2 g/d (95% CI, −203.8 to −78.6) and −306.3 g/d (95% CI, −359.5 to −216.7), respectively, compared with the No-LCS group (Table 2).

Water intake in the No-LCS group increased by 28.4 g/d (95% CI, 23.4–33.5) annually, while the intake of minimally processed foods and beverages rose by 18.2 g/d (95% CI, 11.0–25.3) annually over 11 years (Table 2). No significant differences in the trends in water and minimally processed food and beverage intakes were observed between the No-LCS group and the other LCS groups. By 2018–2019, the High-LCS group continued to have lower intakes of both water and minimally processed foods and beverages compared with the No-LCS group (Table 2; Supplementary Table 4).

## 4 Discussion

This nationally representative study of UK adults examined the association between LCS product consumption and intakes of free sugar, total energy, and broader dietary patterns as characterized by the degree of food processing between 2008 and 2019. This study had four notable findings. First, 45.9% of UK adults consumed LCS products in 2008. While this proportion remained stable over 11 years, the overall level of consumption increased due to rising intake in the High-LCS group. Second, there were no significant differences in free sugar intake or total energy intake across groups with different levels of LCS consumption compared with non-consumers throughout the study period. Third, free sugar intake decreased over time, while energy intake remained stable, with no significant differences in these trends between the No-LCS group and the other LCS consumer groups. Fourth, despite similar daily energy intakes across groups, the High-LCS group had a consistently higher energy obtained from ultra-processed foods and beverages compared with non-consumers. In addition, they consumed lower amounts of minimally processed foods and beverages, as well as water than non-consumers of LCS products throughout the study period.

### 4.1 Comparison with previous studies

Temporal changes in population-level consumption of LCS products, and associations between LCS product consumption and free sugar and total energy intakes, have not been previously assessed in adults using individual-level dietary data. The consumption of LCS products among UK adults in this study is comparable to a previous individual-level and pooled cross-sectional analysis from the US, which reported that 47.8% of adults consumed LCS products between 2007 and 2012 (22). Nationally representative cross-sectional data from Australia reported a lower proportion of LCS product consumers with only 18.2% of adult consumers in 2011–2012 (23). Consistent with the findings of our previous study among UK children (24), the proportion of LCS consumers in adults did not significantly change from 2008 to 2019. However, the median LCS product consumption increased among consumers from 132.0 g/d in 2008–2009 to 170.0 g/d in 2018–2019. This finding broadly aligns with a previous ecological study that showed a global increase in quantities of LCS used in packaged foods and beverages, based on country-level data from 2007 to 2019 (12).

Both nationally representative cross-sectional studies from the US and Australia compared only LCS product consumers and non-consumers. The US study found lower total energy and total sugar intake among LCS product consumers compared with non-consumers (22). In contrast, the Australian study found lower free sugar intake among LCS product consumers but no significant differences in total energy intake between groups (23). More recent data from France, although not nationally representative, have reported cross-sectional comparisons among three groups: non-consumers, lower and higher consumers of LCS, categorized using dietary data collected between 2009 and 2022. The authors reported that the mean intake of total sugar was lowest among non-consumers of LCS whereas the mean intake of added sugar was lowest among higher consumers of LCS (31).

The association between LCS consumption and dietary patterns as characterized by the degree of food processing has not been examined in adults apart from a few studies that reported comparisons for a limited selection of food groups. The French study mentioned earlier revealed that higher LCS consumers had a greater percentage of ultra-processed food and beverage consumption but a slightly lower intake of fruit and vegetables, than lower consumers and non-consumers (31). Our study findings were broadly consistent with the previous study showing that the highest LCS consumers had a greater intake of ultra-processed foods and beverages and a lower intake of water and minimally processed foods and beverages compared with non-consumers. Moreover, we found that the consumption of non-LCS ultra-processed foods and beverages did not differ between LCS groups.

Our analysis has also revealed that while the consumption of minimally processed foods and beverages increased from 2008–2009 to 2018–2019, ultra-processed food and beverage consumption remained stable in all LCS groups. This implies that the differences in dietary patterns between LCS groups remained consistent: the highest LCS consumers had lower intakes of water and minimally processed foods and beverages compared with non-consumers of LCS, which may suggest a displacement of some minimally processed food or beverages -such as water- by LCS products in the diets of the highest LCS consumers. These findings underscore the importance of considering the effects of LCS product consumption on dietary patterns according to food processing levels, as evidence indicates that ultra-processed food consumption is linked to obesity and other cardio-metabolic outcomes, even after adjusting for total energy intake and nutrient composition (17). Additionally, future research is warranted to investigate substitution patterns between LCS product consumption and the intakes of water and minimally processed foods and beverages.

In response to growing public health concerns regarding the potential health impacts of LCS consumption, various countries have implemented policies specifically targeting LCS products. For example, the taxation imposed on sweetened beverages have been extended to those containing LCS in European countries such as France, Belgium, and Norway (35). Furthermore, several European countries have adopted the Nutri-Score front-of-pack labeling system, which provides indication for LCS beverages as less nutritious options (36). In Mexico, front-of-pack warning labels have been introduced for the presence of LCS in packaged products (37). The UK currently has no policies specifically targeting LCS products and the existing measures often promote low-calorie sweeteners as a possible substitute for sugar (21). Implementing regulatory measures to limit the use of LCS in foods and beverages in the UK may be needed to complement existing sugar reduction policies and to support improvements in overall dietary patterns.

### 4.2 Strengths and limitations

This study has three main strengths. The study was based on nationally representative dietary data for the UK adult population and examined for the first time the associations between LCS product consumption and a number of key nutrients and dietary components based on the degree of industrial food processing. Additionally, dietary intake was assessed using a 4-day diet diary allowing for detailed dietary data collection and more accurate estimation of participants’ habitual intake. This study also aimed to capture all LCS products, including LCS foods and beverages, by utilizing the detailed food diary in this study.

There are several limitations to this study. First, LCS product consumption may have been underestimated due to the absence of ingredient lists in the NDNS data. Some LCS products might not have been captured if a brand name was not provided or if the generic name or product descriptors did not sufficiently suggest their relevance to LCS. Second, the specific amounts of LCS used in the products were unavailable. Third, due to the serial cross-sectional design of the NDNS, this study cannot assess individual changes in dietary patterns over time. Finally, self-reported dietary intake is subject to under- and mis-reporting and social desirability bias. However, the NDNS employs several methodological approaches to reduce bias, including the use of portion-size estimation tools, standardized protocols, and the collection of biological samples for validation of dietary data. Furthermore, the diet diaries were only accepted if at least 3 days of data were completed to a satisfactory level following review by a trained interviewer.

## 5 Conclusion

Our analysis of nationally representative data found no evidence of significant associations between LCS product consumption and intakes of free sugar and energy among UK adults, both in terms of quantity and trends in consumption. Additionally, our findings suggest that higher consumption of LCS products was associated with greater quantity and energy intake from ultra-processed foods and beverages, along with a more pronounced increasing trend over time. Conversely, it was associated with lower intake of minimally processed foods and beverages including water. These findings emphasize the need for complementary strategies targeting LCS alongside ongoing sugar reduction initiatives. This could include updating national dietary guidelines to address LCS, incorporating LCS into nutrient profiling systems and front-of-pack labels, improving consumer education on the interpretation of food labels, and limiting LCS in institutional settings, including schools. A more comprehensive strategy that addresses dietary patterns overall is urgently needed to rebalance dietary patterns in the UK away from ultra-processed foods.

## Data Availability

The original contributions presented in the study are included in the article/Supplementary material, further inquiries can be directed to the corresponding author.
