# Supplementary material for: Association of low-calorie sweetened product consumption and intakes of free sugar and dietary patterns in UK adults: a national study from 2008 to 2019
Source: Front Nutr. 2026 May 14;13:1797836. doi: 10.3389/fnut.2026.1797836 (PMC13215840; doi:10.3389/fnut.2026.1797836)
Supplement: Supplementary file 1 [file Table_1.docx]

Supplementary Material

**Appendix Table 1: Median consumption of LCS products among UK adults by year of NDNS data collection**

| Levels of LCS food and beverage consumption | Average daily consumption, median (IQR) | | | | | | | | | | | P-value |
| --- | --- | --- | --- | --- | --- | --- | --- | --- | --- | --- | --- | --- |
|  | 2008-2009 | 2009-2010 | 2010-2011 | 2011-2012 | 2012-2013 | 2013-2014 | 2014-2015 | 2015-2016 | 2016-2017 | 2017-2018 | 2018-2019 |  |
| Low-calorie sweetened food and beverage consumption (g/day), median (IQR) | | | | | | | | | | | |  |
| All LCS consumers | 132.0  (236.2) | 144.8  (288.2) | 150.0  (275.0) | 157.0  (283.9) | 150.0  (299.3) | 146.5  (302.9) | 157.5  (300.0) | 165.0  (330.3) | 157.5  (308.2) | 207.8 (420.0) | 170.0  (336.6) | <0.01** |
| Low-LCS | 40.0  (48.3) | 37.5  (52.3) | 46.3  (37.5) | 43.0  (50.0) | 43.8  (41.3) | 41.3  (51.3) | 46.3  (42.4) | 43.8  (49.0) | 39.5  (38.1) | 39.2  (47.9) | 37.5  (51.0) | 0.4 |
| Mid-LCS | 139.5  (59.0) | 131.5  (53.8) | 125.5  (70.0) | 128.9  (58.8) | 142.0  (35.5) | 142.0  (75.0) | 125.0  (62.0) | 135.0  (69.5) | 125.0  (63.9) | 126.9  (78.1) | 136.0  (63.5) | 0.1 |
| High-LCS | 400.0  (355.9) | 425.0  (338.8) | 465.5  (367.7) | 423.5 (445.1) | 426.0  (361.2) | 424.3  (337.7) | 452.5  (446.0) | 493.8  (387.5) | 438.7  (445.2) | 500.0  (382.5) | 437.8  (415.0) | 0.02* |
| Low-calorie sweetened food and beverage as percentages of total food and beverage intake (%g_total_/d), median (IQR) | | | | | | | | | | | |  |
| All LCS consumers | 4.8  (9.6) | 5.9  (11.4) | 6.2  (12.4) | 6.4  (12.5) | 6.4  (11.2) | 5.5  (10.1) | 6.3  (13.0) | 6.5  (12.3) | 6.3  (10.6) | 7.8  (14.3) | 6.5 (12.8) | 0.02* |
| Low-LCS | 0.1  (1.9) | 1.4  (1.9) | 1.7  (1.7) | 1.8  (2.1) | 1.7  (1.6) | 1.5  (2.0) | 1.9  (2.0) | 1.5  (2.0) | 1.4  (1.7) | 1.4  (1.7) | 1.3  (1.8) | 0.6 |
| Mid-LCS | 5.0  (3.3) | 5.1  (3.4) | 5.2  (3.4) | 5.0  (2.9) | 5.8  (2.7) | 5.1  (3.4) | 4.8  (2.8) | 5.1  (3.3) | 4.9  (2.7) | 4.9  (3.2) | 4.9  (3.0) | 0.6 |
| High-LCS | 15.8  (13.2) | 17.6  (14.0) | 18.4  (15.4) | 17.7  (14.9) | 15.7  (14.0) | 15.4  (13.4) | 19.2  (16.0) | 16.5  (15.7) | 14.9  (15.0) | 16.8  (15.6) | 17.5  (15.1) | 0.7 |

**Statistical analysis:** Median LCS product consumption among all LCS consumers across years was compared using Kruskal-Wallis test. Levels of LCS product consumption across years were compared using quantile regression.

**Abbreviations:** LCS products, no- and low-calorie sweetened products

**Levels of LCS product consumption:** No-LCS, no LCS product consumption (0 gram/day); Low-LCS, daily LCS product consumption ≤ 75.0 grams; Mid-LCS, daily LCS product consumption >75.0-216.8 grams; High-LCS, average daily LCS product consumption >216.8 grams

**Appendix Table 2: Association between daily LCS product consumption and intakes of dietary components from 2008-2019 (n = 8304)**

| Intake of nutrients | Total sugar intake  (g/d) | Energy from ultra-processed foods and beverages  (kcal/d) | Energy from minimally processed foods and beverages  (kcal/d) |
| --- | --- | --- | --- |
|  | Coefficient (95% CI) | Coefficient (95% CI) | Coefficient (95% CI) |
| LCS product consumption at year 1**^+^**  - No-LCS  - Low-LCS  - Mid-LCS  - High-LCS | Reference  8.4 (3.5,13.2)*  0.8 (-4.5, 6.1)  -2.1 (-6.9, 2.8) | Reference  56.0 (9.9, 102.1)*  60.8 (10.9, 110.8)*  119.2 (73.2, 165.2)*^+^ | Reference  4.1 (-24.5, 32.6)  -28.8 (-59.7, 2.2)  -40.4 (-68.9, -11.8)* |
| Survey year | -1.2 (-1.5, -0.8)*^+^ | -1.6 (-5.3, 2.1) | -2.7 (-5.1, -0.5)* |
| Interaction  - No-LCS x Year  - Low-LCS x Year  - Mid-LCS x Year  - High-LCS x Year | Reference  -0.6 (-1.5, 0.2)  -0.3 (-1.2, 0.6)  0.4 (-0.4, 1.2) | Reference  -0.2 (-8.1, 7.8)  -0.3 (-8.7, 8.1)  -2.5 (-9.9, 4.9) | Reference  3.0 (-2.0, 7.8)  -0.4 (-5.6, 4.9)  0.5 (-4.1, 5.0) |

* P-value < 0.05 before Bonferroni correction, *^+^ P-value < 0.05 after Bonferroni correction

**^+^** The levels of LCS product consumption represent nutritional outcome in adults relative to No-LCS in 2008-2009.

**Statistical analysis:** Multivariable linear regression adjusted for age, sex, ethnicity, household income, Indices of Multiple Deprivation, body mass index, and smoking status

**LCS product consumption at Year 1:** Values represent estimated nutritional outcomes for each LCS consumption group relative to the No-LCS group in 2008–2009; **Survey year:** represents the change in the outcome over time in the No-LCS group; **Interaction term (LCS consumption group × survey year):** represents the difference in change over time between each LCS group and the No-LCS group.

**Abbreviations:** LCS product, no- and low-calorie sweetened product; g, grams

**Levels of LCS product consumption:** No-LCS, no LCS product consumption (0 gram/day); Low-LCS, daily LCS product consumption ≤ 75.0 grams; Mid-LCS, daily LCS product consumption >75.0-216.8 grams; High-LCS, average daily LCS product consumption >216.8 grams

**Appendix Table 3: Sugar and energy intake by LCS product consumption levels among UK adults (2008-2009 and 2018-2019)**

| Intake of Energy and sugar | Free sugar intake  (g/d) | | Free sugar intake  (%kcal_total_/d) | | Total energy intake  (kcal/d) | | Total sugar intake  (g/d) | |
| --- | --- | --- | --- | --- | --- | --- | --- | --- |
|  | Year 1  Coefficient (95% CI) | Year 11  Coefficient (95% CI) | Year 1  Coefficient (95% CI) | Year 11  Coefficient (95% CI) | Year 1  Coefficient (95% CI) | Year 11  Coefficient  (95% CI) | Year 1  Coefficient (95% CI) | Year 11  Coefficient  (95% CI) |
| - No-LCS  - Low-LCS  - Mid-LCS  - High-LCS | Reference  4.7  (0.1, 9.3)*  -2.1  (-7.1, 2.8)  -0.3  (-4.9, 4.2) | Reference  -3.9  (-8.3, 0.5)  -1.5  (-6.0, 3.0)  -1.8  (-5.7, 2.0) | Reference  1.3  (0.6, 2.0)*^+^  -0.1  (-0.9, 0.7)  -0.4  (1.2, -0.04) | Reference  -1.4  (-2.2, -0.7)*^+^  -0.3  (-1.1, -0.5)  -0.7  (-1.4, -0.1)* | Reference  36.2  (-22.2, 94.5)  -27.1  (-90.4, 36.1)  14.0  (-44.3, 72.3) | Reference  83.3  (21.8, 144.7)*^+^  2.0  (-60.1, 65.9)  5.0  (-48.9, 59.0) | Reference  8.4  (3.5,13.2)*  0.8  (-4.5, 6.1)  -2.1  (-6.9, 2.8) | Reference  2.1  (-3.1, 7.2)  -2.1  (-7.3, 3.2)  1.9  (-2.6, 6.5) |

* P-value < 0.05 before Bonferroni correction, *^+^ P-value < 0.05 after Bonferroni correction

**Statistical analysis:** Multivariable linear regression adjusted for age, sex, ethnicity, household income, Indices of Multiple Deprivation, body mass index, and smoking status

**LCS product consumption at Year 1:** Since the Survey year variable was centred at Year 1, these estimates provide the average difference in outcome for each LCS consumption group compared with the No-LCS group in 2008–2009 (Year 1); **Survey year:** presents the trend or annual change in outcome over time for the No-LCS group; **Interaction term (LCS consumption group × survey year):** assesses the difference in trends for each LCS consumption group compared with the No-LCS group.

**Abbreviations:** LCS product, no- and low-calorie sweetened product; kcal, kilocalorie; ; g, grams

**Levels of LCS product consumption:** No-LCS, no LCS product consumption (0 gram/day); Low-LCS, daily LCS product consumption ≤ 75.0 grams; Mid-LCS, daily LCS product consumption >75.0-216.8 grams; High-LCS, average daily LCS product consumption >216.8 grams

**Appendix Table 4: Dietary components by LCS product consumption levels among UK adults (2008-2009 and 2018-2019)**

| Intake of dietary components | Ultra-processed food and beverage intake  (g/d) | | Minimally processed food and beverage intake  (g/d) | | Water intake  (g/d) | |
| --- | --- | --- | --- | --- | --- | --- |
|  | Year 1  Coefficient  (95% CI) | Year 11  Coefficient  (95% CI) | Year 1  Coefficient  (95% CI) | Year 11  Coefficient  (95% CI) | Year 1  Coefficient  (95% CI) | Year 11  Coefficient  (95% CI) |
| - No-LCS  - Low-LCS  - Mid-LCS  - High-LCS | Reference  104.3  (59.8, 148.9)*^+^  164.5  (116.1, 212.8)*^+^  545.3  (500.8,589.8)*^+^ | Reference  38.3  (-8.6, 85.2)  148.0  (100.0, 196.1)*^+^  649.4  (608.3, 690.6)*^+^ | Reference  -52.0  (-141.8, 37.8)  -152.2  (-249.5, -54.9)*  -306.3  (-395.9, -216.7)*^+^ | Reference  212.5  (118.0, 307.0)*^+^  -28.7  (-125.5, 68.1)  -240.4  (-323.2, -157.6)*^+^ | Reference  -58.5  (-121.2, 4.2)  -114.5  (-182.4, -46.5)*  -141.2  (-203.8, -78.6)*^+^ | Reference  55.5  (-10.6, 121.6)  -10.5  (-78.1, 57.1)  -194.2  (-252.1, -136.3)*^+^ |

| Intake of dietary components as percentages of total energy intake | Energy from ultra-processed foods and beverages  (kcal/day) | | Energy from minimally processed foods and beverages  (kcal/day) | |
| --- | --- | --- | --- | --- |
|  | Year 1  Coefficient  (95% CI) | Year 11  Coefficient  (95% CI) | Year 1  Coefficient  (95% CI) | Year 11  Coefficient  (95% CI) |
| - No-LCS  - Low-LCS  - Mid-LCS  - High-LCS | Reference  56.0  (9.9, 102.1)*  60.8  (10.9, 110.8)*  119.2  (73.2, 165.2)*^+^ | Reference  54.1  (5.6, 102.6)*  57.6  (7.9, 107.3)*  94.0  (51.5, 136.5)*^+^ | Reference  4.1  (-24.5, 32.6)  -28.8  (-59.7, 2.2)  -40.4  (-68.9, -11.8)* | Reference  33.6  (3.5, 63.7)*  -32.3  (-63.1, -1.5)*  -35.8  (-62.1, -9.4)* |

* P-value < 0.05 before Bonferroni correction, *^+^ P-value < 0.05 after Bonferroni correction

**^+^** The levels of LCS product consumption represent nutritional outcome in adults relative to No-LCS in 2008-2009.

**Statistical analysis:** Multivariable linear regression adjusted for age, sex, ethnicity, household income, Indices of Multiple Deprivation, body mass index, and smoking status

**Abbreviations:** kcal, kilocalories LCS product, no- and low-calorie sweetened product

**Levels of LCS product consumption:** No-LCS, no LCS product consumption (0 gram/day); Low-LCS, daily LCS product consumption ≤ 75.0 grams; Mid-LCS, daily LCS product consumption >75.0-216.8 grams; High-LCS, average daily LCS product consumption >216.8 grams

**
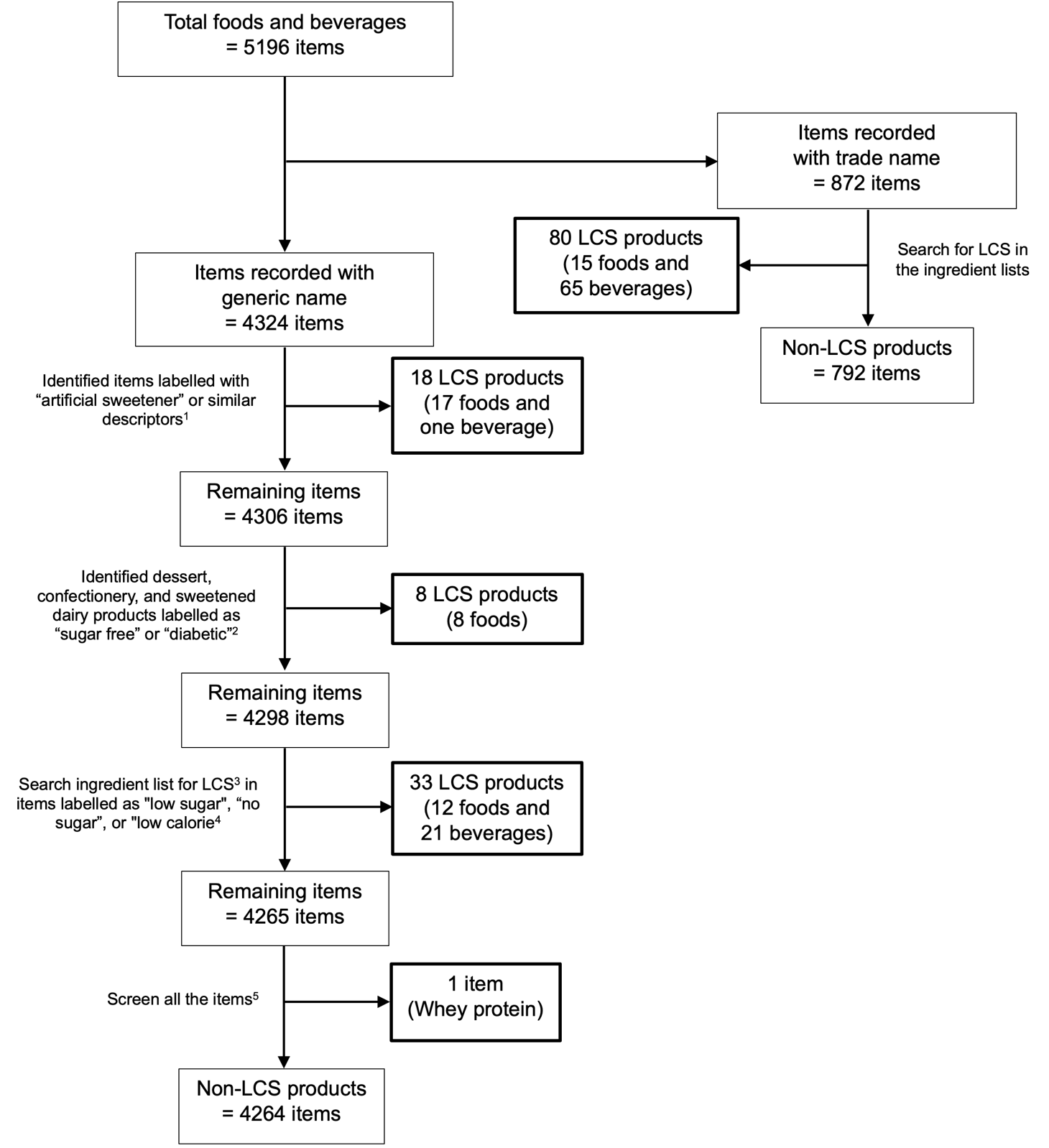
**

**Appendix Figure 1: Flow chart for the identification of LCS products in the National Diet and Nutrition Survey**

^1^e.g., artificial sweetener, milkshake with artificial sweetener”, and “potato crisps with artificial sweetener”

^2^e.g., sweetened yogurt, chocolate, custard, and candy

^3^Ingredients for these products were searched and reviewed. Items were classified as LCS products if any brand on the market contained LCS.

^4^e.g., salad dressing, sauce, soup, carbonated drinks, and juice drinks

^5^All remaining packaged products had their ingredient lists searched, and they were classified as LCS products if any brand available on the market contained LCS.

**
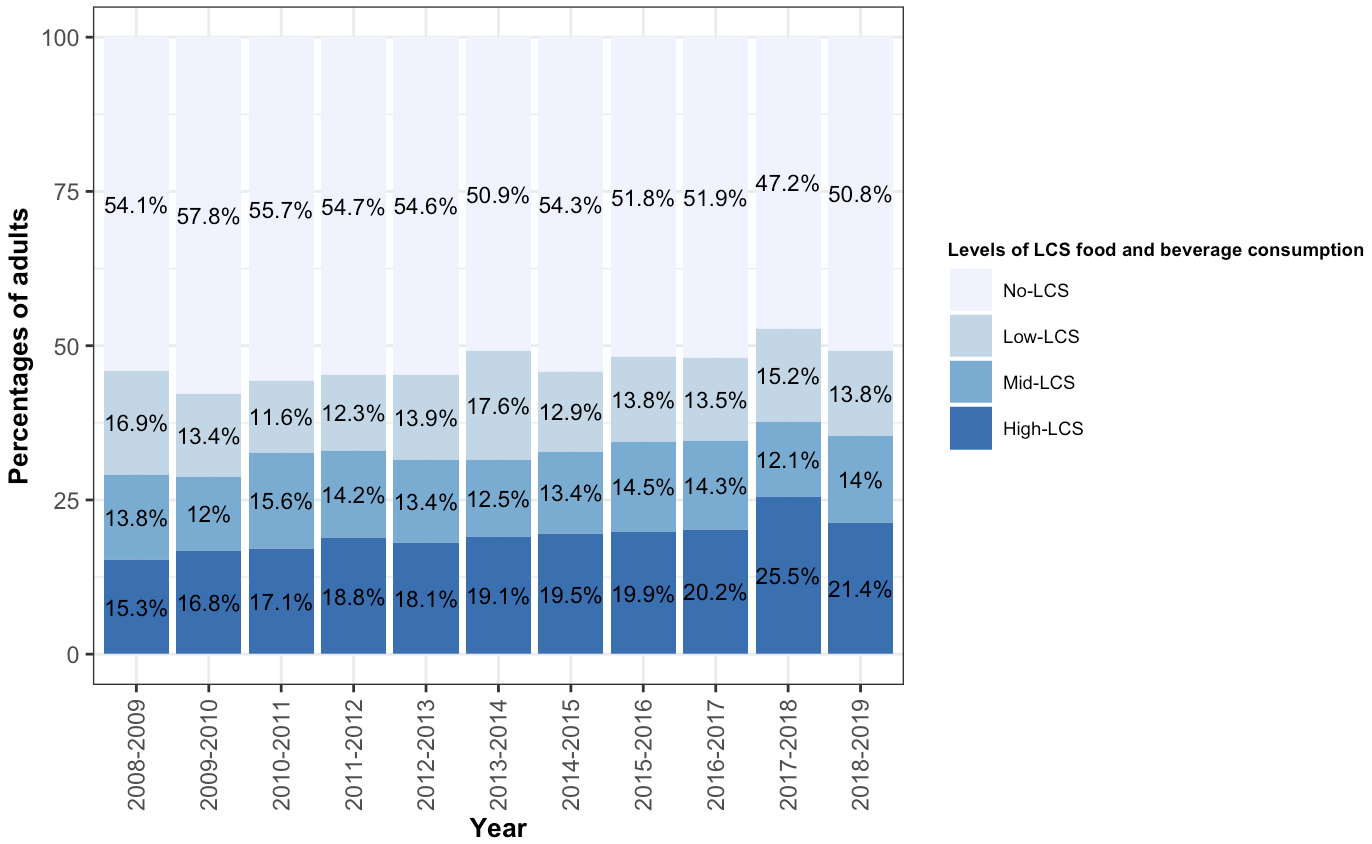
**

**Appendix Figure 2: Trends in proportion of UK adults across levels of LCS product consumption from 2008-2019**

54.1%

Percentages of adults across level of LCS product consumption were compared using chi-square test: P-value < 0.001

**Abbreviations:** LCS product, no- and low-calorie sweetened product

**Levels of LCS product consumption:** No-LCS, no LCS product consumption (0 gram/day); Low-LCS, daily LCS product consumption ≤ 75.0 grams; Mid-LCS, daily LCS product consumption >75.0-216.8 grams; High-LCS, average daily LCS product consumption >216.8 grams

**
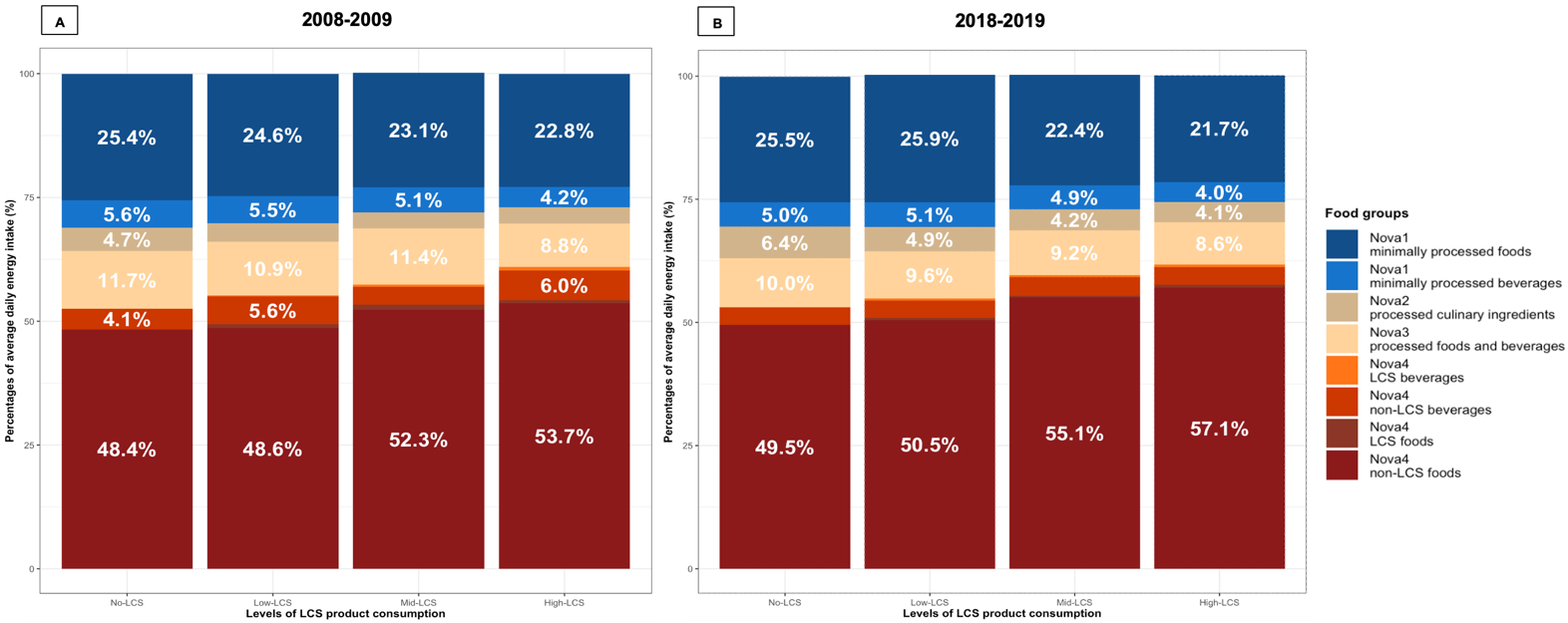
**

**Appendix Figure 3: Daily energy intake by Nova subgroup in 2008-2009 (A) and 2018-2019 (B)**

**Appendix Figure 3A (N = 836)**

**Percentages of energy intake by Nova subgroups**

Low-LCS: Nova 2 processed culinary ingredients, 3.7; Nova 4 LCS beverages, 0.2%; Nova 4 LCS foods, 0.8%

Mid-LCS: Nova 2 processed culinary ingredients, 3.2; Nova 4 LCS beverages, 0.4%; Nova 4 non-LCS beverages, 3.6; Nova 4 LCS foods, 1.0%

High-LCS: Nova 2 processed culinary ingredients, 3.2; Nova 4 LCS beverages, 0.7%; Nova 4 LCS foods 0.6%

**Appendix 3B (N = 585)**

**Percentages of energy intake by Nova subgroups**

No-LCS: Nova 4 non-LCS

beverages; 3.6%

Low-LCS: Nova 4 LCS beverages, 0.4%; Nova 4 non-LCS beverages, 3.5; Nova 4 LCS foods, 0.5%

Mid-LCS: Nova 4 LCS beverages, 0.3%; Nova 4 non-LCS beverages, 3.8; Nova 4 LCS foods, 0.3%

High-LCS: Nova 4 LCS beverages, 0.5%; Nova 4 non-LCS beverages, 3.6; Nova 4 LCS foods 0.6%

**Abbreviations:** LCS product, no- and low-calorie sweetened product; LCS foods, low-calorie sweetened foods; LCS beverages, low-calorie sweetened beverages

**Levels of LCS product consumption:** No-LCS, no LCS product consumption (0 gram/day); Low-LCS, daily LCS product consumption ≤ 75.0 grams; Mid-LCS, daily LCS product consumption >75.0-216.8 grams; High-LCS, average daily LCS product consumption >216.8 grams
